# Treatment durability, satisfaction and quality of life in virologically suppressed HIV‐1 people switching to doravirine: Results from the French study DoraVIH

**DOI:** 10.1111/hiv.70210

**Published:** 2026-02-19

**Authors:** Bruno Spire, Olivier Robineau, Valérie Pourcher, Paul Loubet, Christia Palacios, Christine Jacomet, Haydar Benachir, Philippe Mariot, Jean‐Jacques Parienti, Laurence Slama

**Affiliations:** ^1^ Aix Marseille University, Inserm, IRD, SESSTIM Economic & Social Sciences of Health & Medical Information Processing, ISSPAM Marseille France; ^2^ CH Tourcoing Tourcoing France; ^3^ AP‐HP Pitié Salpêtrière Paris France; ^4^ CHU Nîmes Nîmes France; ^5^ AP‐HP Tenon Paris France; ^6^ CHU Clermont Ferrand Clermont Ferrand France; ^7^ MSD France Puteaux France; ^8^ Caen University Hospital Caen France; ^9^ AP‐HP Hôtel‐Dieu Paris France

**Keywords:** doravirine, HIV, patient‐reported outcomes, real world, switch

## Abstract

**Objectives:**

In Europe, most people with HIV‐1(PWH) achieve virologic suppression with effective and well‐tolerated antiretroviral therapies (ART). In this context, patient‐reported outcomes (PRO) are increasingly important for evaluating ART benefits. This study aimed to describe treatment durability, satisfaction and quality of life (QoL) in virologically suppressed PWH switching to a doravirine‐based regimen (PWH‐DOR).

**Methods:**

DoraVIH was a national, multicentre, real‐world cohort study with two phases: cross‐sectional and prospective follow‐up of PWH‐DOR at 3 (M3) and 15–18 months (M15–18) post‐switch. Durability of the doravirine‐based regimen was recorded at both follow‐up visits, and on‐treatment virologic response at M3. QoL was assessed at baseline (D0) and M15–18 using the SF‐36 questionnaire. Pre‐switch patient satisfaction with treatment was assessed at D0 using the HIV Treatment Satisfaction Questionnaire (HIVTSQs), and post‐switch at M3 and M15–18 using the HIVTSQc.

**Results:**

Among 143 PWH‐DOR included (mean age 51.1 ± 10.6 years; 68% men), durability was 94% (131/140) at M3 and 83% (114/138) at M15–18. All participants receiving doravirine at M3 remained virologically suppressed. SF‐36 scores showed no significant QoL change between D0 (72.6 ± 17.2) and M15–18 (73.0 ± 19.6). Pre‐switch treatment satisfaction averaged 50.2 ± 9.6 (HIVTSQs score). Most PWH‐DOR reported better satisfaction (HIVTSQc score >0): 90% at M3 (96/107) and 90% at M15–18 (89/99).

**Conclusion:**

Despite the absence of a control group and limited sample size, these real‐world data support the effectiveness of switching to a doravirine‐based regimen, with high durability and improved patient satisfaction.

## INTRODUCTION

Considerable progress has been made in developing antiretroviral therapy (ART) regimens that offer improved effectiveness, safety and tolerability. The primary goal of these regimens is to achieve and sustain undetectable viral load, thereby enhancing the life expectancy of people with HIV [1]. Therefore, most people with HIV receiving ART regimens are virologically suppressed. In this context, quality of life (QoL) and treatment satisfaction are increasingly important for choosing ART regimens.

Switching can improve health status and QoL of people with HIV. However, limited research has been conducted to explore regimen switching in the real world, as most clinical trials are conducted in controlled environments [2, 3, 4]. This may not accurately reflect the diversity of participants and variability in physicians' treatment decisions. In real‐world settings, a wide range of adherence patterns and different clinical outcomes may be observed compared to those in controlled clinical trials [5, 6, 7], potentially providing a more comprehensive understanding of patients' treatment preferences and physician decision‐making processes.

The impact of treatment switching on quality of life likely requires trials specifically designed to assess these dimensions prospectively and with minimal bias. The DoraVIH study looked into this in standard practice in France. This national, observational, multicentric, two‐step real‐world longitudinal study combined a cross‐sectional study at baseline and a focused prospective follow‐up cohort. The first cross‐section phase demonstrated that the main reasons for switching ART were treatment simplification and improved tolerance [8]. Participants with obesity were more likely to switch to doravirine, reflecting physicians' favorable perception of the potential benefit of doravirine, particularly in managing weight gain [9]. Doravirine has demonstrated safety and tolerability in both naive and pretreated people with HIV [10, 11, 12, 13], with a low rate of discontinuation due to adverse events in clinical trials [10]. Switch to a doravirine‐based regimen has proven to be a well‐tolerated option for virologically suppressed people with HIV considering a therapy change, maintaining high rates of virologic suppression [13].

The present work is based on the second longitudinal phase of the DoraVIH study. The objective was to describe QoL as well as treatment satisfaction and durability of the people with HIV switching to doravirine‐based regimen, by comparing data obtained at baseline (before switch) and those obtained during the follow‐ups.

## MATERIALS AND METHODS

### Ethics

DoraVIH protocol was approved by the competent national ethics committee (Committee for Personal Protections of Ile de France X, authorization number 85–2021) prior to enrolment of study participants. The non‐opposition to participation of all patients was documented in their medical files, in accordance with French law. This study was conducted in accordance with the Good Pharmacoepidemiology Practice and the Declaration of Helsinki.

### Design and study population

DoraVIH was a French, multicentric (15 sites), real‐world cohort study that used a two‐step analytical approach. The first step consisted of a cross‐sectional baseline assessment describing the reasons for switching (primary objective) [8]. The second step involved a focused prospective follow‐up cohort designed to address the secondary objectives related to doravirine‐based regimens.

Adults (aged ≥18 years) with HIV virologically suppressed (plasma HIV‐1 RNA of <50 copies/mL) for at least 6 months prior to inclusion and on a stable ART (any HIV drugs available in France) were included. None had prior exposure to doravirine. Pregnant women were excluded. The target convenient sample size was 300 participants.

Demographic characteristics, medical and HIV histories, and ART switch details were collected at baseline (D0) for all participants. Depending on the ART regimen started at D0, participants were divided into two groups of approximately equal sizes: those who switched to a doravirine‐based regimen and those who switched to other regimens. Doravirine‐based regimen contained doravirine [1] as a single agent formulation or [2] as a fixed‐dose combination with lamivudine (3TC) and tenofovir disoproxil fumarate (TDF) in a single tablet.

In order to avoid unnecessary burden for both participants and investigators and to ensure feasibility within real‐world settings, patient‐reported outcomes (PRO) and the prospective follow‐up data, supporting the secondary study objectives, were collected on people with HIV switching to a doravirine‐based regimen only. People with HIV switching to a doravirine‐based regimen were followed up at 3 months and between 15 and 18 months after inclusion (M3 and M15–18, respectively). In people with HIV switching to a doravirine‐based regimen, treatment durability was assessed at M3 and M15–18; treatment response at M3; health status at D0 and M15–18; treatment satisfaction at D0, M3 and M15–18.

The cross‐sectional baseline data of all participants were already published [8]. Herein, part of such data was newly analysed to evaluate which baseline factors could potentially predict the durability of treatment at the end of the study (M15–18).

### Instruments

#### Response to treatment

Response to treatment was evaluated at M3 in accordance with clinical practice, based on plasma HIV‐1 RNA levels (copies/mL) being assessed. Patients with a viral load <50 copies/mL were considered virologically suppressed. Therapeutic success or failure was discussed with investigators in the event of blips, defined as intermittently increases above the lower limit of assay detection.

#### Health status

Participant's health status was evaluated at the baseline by the clinician using the Excellent, Very good, Good, Fair or Poor (EVGFP) scale. This rapid 5‐point scale provides an overall clinical appraisal of the patient's health. The EVGFP scale is a measure used in several studies and has shown predictive value for various health outcomes [14, 15].

The Short Form 36 Health Survey questionnaire (SF‐36) was self‐administered at D0 and M15–18. The 36 items/questions composing the SF‐36, once combined, produce eight scales: Physical functioning, Role limitations due to physical health, Pain, General health, Energy/fatigue, Social functioning, Role limitations due to emotional problems and Emotional well‐being. These scales were combined to calculate two main composite scales: Physical Composite Score (PCS), with the first four scales, and Mental Composite Score (MCS), with the final four scales. Together, these two components form the SF‐36 score, ranging from 0 to 100. Higher scores indicate better QoL.

#### Satisfaction with ART

The HIV Treatment Satisfaction Questionnaire (HIVTSQ) was used to measure satisfaction with ART [16, 17].

The status version of the HIVTSQs was self‐administered at D0 to score the satisfaction with the treatment prior to switch. The 10 items scored on a scale from 0 (e.g., very dissatisfied) to 6 (e.g., very satisfied) and were combined to produce a general satisfaction/clinical subscale and a lifestyle/ease subscale, both ranging from 0 to 30. Subscales were summed to produce a treatment satisfaction score ranging from 0 to 60. The higher the score, the greater the satisfaction with the treatment prior to switch.

The change version the HIVTSQc was self‐administered at M3 and M15–18 to access changes in satisfaction with ART following the switch. The 10 items scored on a scale from −3 (e.g., much less satisfied) to 3 (e.g., much more satisfied) and were combined to produce two subscales ranging from −15 to 15. Subscale scores were summed to produce a relative treatment satisfaction score ranging from −30 to 30. The higher the score, the greater the improvement in satisfaction with treatment; the lower the score, the greater the deterioration in satisfaction in treatment. A score of 0 represents no change.

### Statistical methods

We first described the population using descriptive statistics including frequencies and percentages for categorical endpoints; mean and standard deviation (SD), confidence interval (CI) 95%, median, interquartile range (IQR) and range for continuous variables.

We then compared variables at baseline and at the endpoints using Chi‐square or Fisher's exact test for categorical variables and using Student's *t*‐test or Wilcoxon–Mann–Whitney test, as appropriate for continuous variables. A *p*‐value <0.05 was considered statistically significant, without adjustments for multiple comparisons due to the exploratory nature of the study.

Baseline factors were analysed to predict the durability of the doravirine‐based regimen at the M15–18 follow‐up. Univariable regressions were run for a predetermined set of covariates in order to identify significant factors for inclusion in a subsequent multivariable regression (factors with a *p* ≤ 0.2 are kept for multivariable regressions).

Statistical analyses were conducted using SAS software version 9.4 (Copyright© 2016 by SAS Institute Inc., Cary, NC, USA).

## RESULTS

The study population consisted of long‐term treated people with HIV (virologically suppressed at least 6 months prior to inclusion and on a stable ART). Between December 13, 2021, and September 21, 2022, a total of 291 people with HIV were included and analysed in the DoraVIH study. Of these, 143 (49.1%) switched to a doravirine‐based regimen: 122 (85.3%) switched to a fixed‐dose combination in a single tablet (DOR/3TC/TDF), and 21 (14.7%) to a single agent formulation.

At baseline (Table 1), the mean age of people with HIV switching to a doravirine‐based regimen was 51.1 ± 10.6 years, and 97 participants (67.8%) were men. The main socio‐professional category was employees for 62 participants (43.4%), and the majority (*N* = 96, 67.1%) were beneficiaries of complementary health insurance (Individual, Collective or Solidarity fund categories grouped). The body mass index (BMI) was ≥30 kg/m^2^ for 35 participants (25.0%). The presence of one, two or more of predefined medical conditions were indicated for 89 (62.2%) people with HIV switching to a doravirine‐based regimen, and 106 participants (74.1%) had at least one concomitant medication. Regarding the history of HIV infection, median duration from diagnosis of HIV infection to D0 was 17.3 years, 14.3 years from date of first ART to D0 and 3.5 years from date of previous ART to D0.

**TABLE 1:** Baseline demographic and clinical characteristics of people with HIV switching to a doravirine‐based regimen.

	People with HIV switching to a doravirine‐based regimen (*N* = 143)
Age at inclusion (years)	
Mean (SD)	51.1 (10.6)
Median [Q1–Q3]	50.0 [45.0–58.0]
Min–Max	23–81
Sex, *N* (%)	
Male	97 (67.8)
Female	45 (31.5)
Other	1 (0.7)
Socio‐professional category, *N* (%)	
Employees	62 (43.4)
Unknown	37 (25.9)
Intermediate professions	13 (9.1)
Managers and professionals (high intellect)	16 (11.2)
Craftsmen, traders, business owners	8 (5.6)
Laborers	7 (4.9)
Insurance Status, *N* (%)	
Complementary health insurance (individual ‐ mutual insurance company)	63 (44.1)
State funded	33 (23.1)
Complementary health insurance (Collective)	27 (18.9)
Unknown	14 (9.8)
Complementary health insurance (Solidarity fund)	6 (4.2)
BMI (kg/m^2^)	
Mean (SD)	27.0 (5.4)
Median [Q1–Q3]	26.0 [23.6–29.9]
Min–Max	17.2–45.8
Medical conditions, *N* (%)	
None of the conditions listed	54 (37.8)
1 of the conditions listed	46 (32.2)
2 or more of conditions listed	43 (30.0)
Concomitant medications per patient, *N* (%)	
None	37 (25.9)
1 medication	35 (24.5)
2 medications	21 (14.6)
3 or more medications	50 (35.0)
Time from diagnosis of HIV infection to inclusion (years)	
Mean (SD)	18.1 (10.7)
Median [Q1–Q3]	17.3 [8.2–27.1]
Min–Max	0.9–39.0
Time from date of first ART to inclusion (years)	
Mean (SD)	15.4 (9.0)
Median [Q1–Q3]	14.3 [7.0–23.4]
Min–Max	0.9–39.0
Time on previous ART regimen till switch (years)	
Mean (SD)	4.2 (3.3)
Median [Q1–Q3]	3.5 [2.0–5.0]
Min–Max	0.1–19.0

Of the 143 people with HIV switching to a doravirine‐based regimen, 137 (95.8%) had at least one documented reason for switching ART treatment. The main reasons for ART switching were treatment simplification (*N* = 49, 34.3%), tolerability (*N* = 47, 32.9%) and drug–drug interactions (*N* = 12, 8.4%). The physician was the main initiator of the switch, being responsible for 84 (58.7%) switch initiations. Both the patient and physician were responsible for 41 (28.7%), while patients alone initiated 18 switches (12.6%).

Most of the participants were still being followed at both visits: 140 (97.9%) people with HIV switching to a doravirine‐based regimen at M3, and 138 (96.5%) people with HIV switching to a doravirine‐based regimen at M15–18.

More than 90% of the 140 participants followed at M3 (*N* = 131) were receiving a doravirine‐based regimen as well as 82.6% of the 138 participants followed up at M15–18 (*N* = 114). No baseline factor was associated with durability (Table 2).

**TABLE 2:** Results of the univariable regressions on predetermined set of covariates for explaining DOR‐based regimen durability at M15–18 (*N* = 138 patients under DOR‐based regimen).

Factors predicting durability at M15–18 follow‐up	OR	95% CI	*p*
Sex			0.87
Male versus Female	1.28	0.51; 3.21	
Age at inclusion (years)			0.43
	1.02	0.97; 1.06	
BMI (kg/m^2^)			0.89
<25.0 versus 25.0–29.9	1.25	0.42; 3.76	
≥30.0 versus 25.0–29.9	0.97	0.29; 3.20	
BMI (kg/m^2^)			0.93
	1.00	0.92; 1.09	
Obesity (BMI ≥30 kg/m^2^)			0.76
Obese versus Non‐obese	0.85	0.30; 2.40	
Insurance status (grouped)			0.65
Complementary health insurance versus State funded	0.98	0.35; 2.74	
Unknown versus State funded	2.67	0.29; 24.63	
Socio‐professional Category			0.83
Employees versus Craftsmen, traders, business owners	0.89	0.09; 8.41	
Intermediate professions versus Craftsmen, traders, business owners	0.45	0.04; 5.21	
Laborers versus Craftsmen, traders, business owners	1.20	0.06; 24.47	
Managers and professionals (high intellect) versus Craftsmen, traders, business owners	1.30	0.10; 17.73	
Unknown versus Craftsmen, traders, business owners	1.28	0.12; 13.35	
Time from diagnosis of HIV infection to inclusion (years)			0.69
	1.01	0.97; 1.05	
Time from date of first ART to inclusion (years)			0.45
	1.02	0.97; 1.07	
Time on previous ART regimen till switch (years)			0.55
	1.05	0.90; 1.22	
Overall Health Assessment (grouped)			0.93
Excellent versus Good	0.91	0.28; 2.97	
Fair or poor versus Good	>9999.99	<0.01; >9999.99	
Very good versus Good	1.36	0.46; 4.03	
Doravirine formulation			0.74
	1.23	0.37; 4.05	
SF‐36 total score			0.22
	1.02	0.99; 1.04	
HIVTSQs total score			0.38
	1.02	0.98; 1.07	
Initiator of switch			0.16
Both versus Patient	1.44	0.40; 5.16	
Physician versus Patient	2.96	0.86; 10.18	
Reason of switch			
Prior treatment failure			0.99
	>9999.99	<0.01; >9999.99	
Drug–drug interactions			0.94
	1.06	0.22; 5.17	
Tolerability			0.26
	0.59	0.24; 1.47	
Financial			0.99
	>9999.99	<0.01; >9999.99	
Treatment simplification			0.16
	0.52	0.21; 1.28	
Pregnancy			0.99
	<0.01	<0.01; >9999.99	
Other			0.34
	1.68	0.58; 4.87	

### Response to treatment

Among the 131 patients still receiving doravirine‐based regimen at M3, viral suppression (plasma HIV‐1 RNA levels <50 copies/mL) was confirmed in 122 (93.1%) people with HIV switching to a doravirine‐based regimen. Viral load information was missing for seven (5.3%) participants. Of these, six participants were receiving doravirine‐based regimen at the end of the study (M15–18), and the seventh patient withdrew consent before reaching M15–18. Finally, only two patients (1.5%) presented plasma HIV‐1 RNA levels >50 copies/mL (65 and 355 copies/mL). These cases, corresponding to blips, were not considered therapeutic failures by the investigators and did not lead to a change in ART. Therefore, no confirmed virologic failure was observed.

### Health status

At D0, the EVGFP was completed by physician for 129 (90.2%) people with HIV switching to a doravirine‐based regimen, being rated as good (45.0%), very good (32.6%) or excellent (19.4%) for 125 of them (96.9%), and poor for only two participants. The EVGFP scores reported by physicians were cross‐tabulated with the SF‐36 scores from patients at D0. As expected, high mean of SF‐36 scores was observed in patients whose overall health was stated by physicians as excellent (77.8 ± 15.8), very good (72.8 ± 16.2) and good (69.7 ± 18.4) and a low mean of SF‐36 total score (50.5 ± 13.2) was found in patients that received fair or poor statement from physicians.

The SF‐36 questionnaire was completed by 97.2% (*N* = 139) people with HIV switching to a doravirine‐based regimen at D0 and by 86.2% (*N* = 119) of the 138 participants that completed the M15–18 follow‐up. A total of 117 participants completed the SF‐36 questionnaire at both D0 and M15–18. Of these, 83.8% (*N* = 98) were receiving a doravirine‐based regimen at M15–18 follow‐up. For these participants, SF‐36 mean score was 72.6 ± 17.2 at D0 and 73.0 ± 19.6 at M15–18. There is no significant difference between the two visits regarding the SF‐36 score (*p* = 0.68).

**FIGURE 1:**
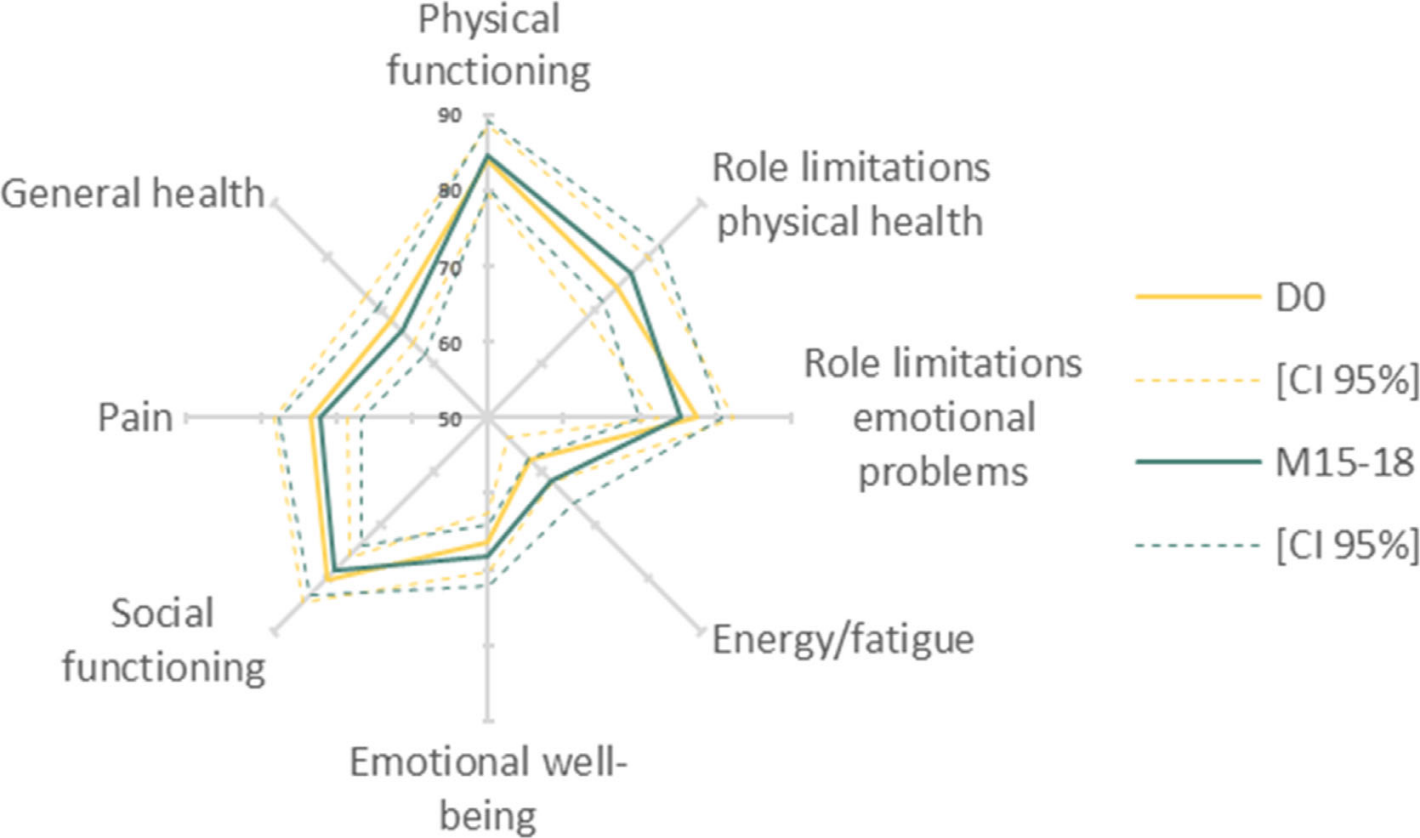
Evolution of SF‐36 scales in participants receiving doravirine‐based regimen. The inclusion visit (D0) is indicated with a yellow line and the M15–18 follow‐up is indicated with a green line. Discontinuous lines represent the 95% confidence interval. On each axis, SF‐36 scales are shown. The numerical scale represents the score obtained in each scale of the SF‐36 questionnaire. The value indicated in the upper part of the scale represents the maximal score recorded. *N* = 98, participants with SF‐36 score and receiving doravirine‐based regimen at both D0 and M15–18.

Figure 1 presents the mean score of the eight SF‐36 scales at D0 and M15–18 of people with HIV switching to a doravirine‐based regimen.

### Treatment satisfaction

Most people with HIV switching to a doravirine‐based regimen completed the HIVTSQ at D0 (*N* = 138/143, 96.5%). Of the 140 participants still being followed at M3, 112 (80.0%) completed the HIVTSQ, including 107 (95.5%) receiving a doravirine‐based regimen. Of the 138 participants still being followed at M15–18, 112 (81.2%) completed the HIVTSQ, including 99 (88.4%) receiving a doravirine‐based regimen.

Results of HIVTSQs/c in people with HIV switching to a doravirine‐based regimen are shown in Table 3. At D0, the mean HIVTSQs score (0 to 60) was 50.2 ± 9.6, ranging from 13.0 to 60.0 with a median of 53.0, indicating a high level of treatment satisfaction prior to the switch. At M3, the mean HIVTSQc score (−30 to 30) was positive (18.4 ± 11.2), with a CI 95% (16.3; 20.5). Since the CI does not contain the score 0, this represents a clear improvement in satisfaction after switching ART. At M15–18, similar results were observed with a higher mean HIVTSQc score (20.5 ± 11.0). However, CI 95% overlapped at the two post‐switching time points.

**TABLE 3:** Details of HIVTSQ in participants receiving doravirine‐based regimen.

	Inclusion visit D0	Follow‐up visit M3	Follow‐up visit M15–18
	(*N* = 138)	(*N* = 107)	(*N* = 99)
HIVTSQs/c score^a^			
Mean (SD)	50.2 (9.6)	18.4 (11.2)	20.5 (11.0)
CI 95%	48.6; 51.9	16.3; 20.5	18.3; 22.7
Median [Q1–Q3]	53.0 [45.0 to 58.0]	22.0 [10.0 to 29.0]	25.0 [15.0 to 29.0]
Min–Max	13.0–60.0	−15.0 to 30.0	−23.0 to 30.0
General Satisfaction/Clinical subscale^b^			
Mean (SD)	24.7 (5.6)	9.2 (5.9)	10.4 (5.9)
CI 95%	23.8; 25.6	8.1; 10.4	9.2; 11.5
Median [Q1–Q3]	26.0 [21.0 to 30.0]	11.0 [4.0 to 15.0]	13.0 [8.0 to 15.0]
Min–Max	2.0–30.0	−12.0 to 15.0	−13.0 to 15.0
Lifestyle/Ease subscale^b^			
Mean (SD)	25.5 (4.7)	9.2 (5.8)	10.1 (5.5)
CI 95%	24.7; 26.3	8.0; 10.3	9.0; 11.2
Median [Q1–Q3]	27.0 [23.0 to 30.0]	11.0 [4.0 to 15.0]	12.0 [6.0 to 15.0]
Min–Max	10.0–30.0	11 (10.3)	−10.0 to 15.0
Patients with increased satisfaction, *N* (%)			
Increased satisfaction^c^		96 (89.7)	89 (89.9)
Decreased/similar satisfaction^d^		11 (10.3)	10 (10.1)

^a^ D0: HIVTSQs absolute score from 0 to 60; M3 & M15–18: HIVTSQc relative score from −30 to 30.

^b^ D0: HIVTSQs absolute subscales from 0 to 30; M3 & M15–18: HIVTSQc relative subscales from −15 to 15.

^c^ Total HIVTSQc score >0.

^d^ Total HIVTSQc score ≤0.

**FIGURE 2:**
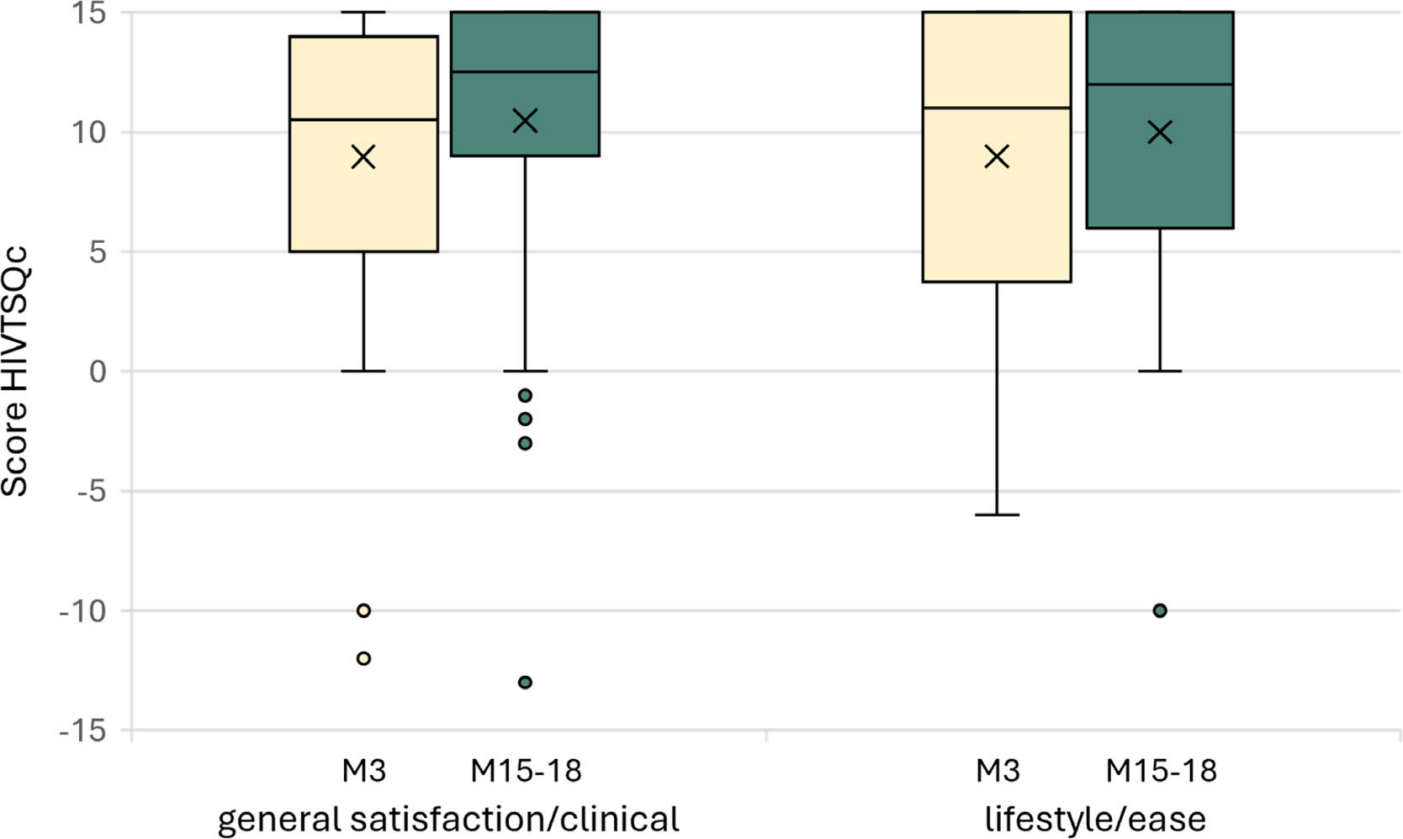
Distribution of HIVTSQc subscales differences from baseline in participants receiving doravirine‐based regimen at M3 and M15–18 follow‐ups. Box (median and Q1‐Q3) and whisker (minimum and maximum) plots represent HIVTSQc subscales (general satisfaction/clinical and lifestyle/ease) at M3 (yellow) and M15–18 (green) follow‐ups. Mean is indicated by X and outliers by dots. HIVTSQc was completed by 107 participants at M3 and 99 participants at M15–18 receiving a doravirine‐based regimen.

The Figure 2 presents the distributions of HIVTSQc subscales (general satisfaction/clinical and lifestyle/ease) at M3 and M15–18. Almost 90% of the people with HIV switching to a doravirine‐based regimen related increased satisfaction with their doravirine‐based regimen at both M3 and M15‐18 (HIVTSQc score >0). There were only two (1.9%) participants with a negative score at M3 and three (3.0%) at M15–18.

## DISCUSSION

In the present article, we showed the results obtained with the prospective follow‐up of participants of the DoraVIH study, conducted in France. People with HIV who switched to a doravirine‐based regimen were followed up 3 and between 15 and 18 months after switch. Data were collected on treatment durability, QoL and treatment satisfaction in real‐world settings.

People with HIV switching to a doravirine‐based regimen sample is briefly described in this article, as a full description of the study participants has already been published with findings from the cross‐sectional section [8].

Real‐life data on doravirine use are limited [18, 19], and as far as we know, this is the first study reporting treatment satisfaction of HIV virologically suppressed people with HIV after ART switch to a doravirine‐based regimen in real‐world settings in France. Key findings of this study include a high rate of treatment durability and response to treatment (no confirmed virologic failures), as well as increased treatment satisfaction of patients receiving a doravirine‐based regimen.

A substantial number of patients was receiving doravirine at the end of the study: 93.6% of people with HIV switching to a doravirine‐based regimen at M3, and 82.6% at M15–18. However, it was not possible to infer the reasons for this durability, as none of the baseline factors predicted doravirine‐based regimen durability.

As expected [13, 20], the response to treatment was high, with no confirmed virologic failures reported at M3. It is well established that achieving and maintaining virologic suppression in people with HIV is closely related to adherence to ART, which, in turn, depends on patient treatment satisfaction. Aligned with this, we observed an improvement in treatment satisfaction among people with HIV switching to a doravirine‐based regimen, being reported by almost 90% of participants under doravirine during the two follow‐up assessments conducted at M3 (89.7%) and M15–18 (89.9%).

The high treatment‐satisfaction scores observed in our cohort are likely attributable, at least in part, to treatment simplification, as most people with HIV switching to a doravirine‐based regimen switched to a fixed‐dose combination of doravirine, 3TC and TDF in a single tablet (79.6%), a formulation known to reduce pill burden and improve convenience [21]. This is consistent with findings from a cross‐sectional multicentre study conducted in Japan with 679 patients, whose results suggest that switching to a single‐tablet once‐daily regimen improves HIVTSQ scores in patients receiving twice‐ or thrice‐daily regimens [22]. High patient satisfaction with doravirine was also demonstrated in a retrospective multicentre cohort study conducted in Italy with 95 patients who switched to a doravirine‐based regimen associating integrase strand inhibitors (raltegravir or dolutegravir) [23]. High patient satisfaction observed in the Italian cohort may also reflect the favourable tolerability profile of doravirine, as the study reported good overall tolerability and a low rate of adverse events leading to discontinuation.

Interestingly, high treatment‐satisfaction scores were also reported among people with HIV who had stopped the doravirine‐based regimen at M15–18. Regardless of the small sample size and although reasons for discontinuing treatment and details of subsequent ART were not collected, this finding may reflect an improvement in participant satisfaction with treatment, irrespective of subsequent regimen changes. Moreover, it highlights the potential enhancement in therapeutic management resulting from the switch.

Regarding participants' health status, no significant differences were found between mean scores of the SF‐36 questionnaire at baseline and at M15–18. This may reflect the limited sensitivity of the SF‐36 scale in detecting subtle quality‐of‐life variations in people with HIV. Although it is one of the most commonly used QoL instruments [24], the SF‐36 indeed may not adequately detect nuanced quality‐of‐life issues in clinically stable people with HIV due to ceiling effects, its generic design, limited sensitivity to HIV‐related changes in HIV‐specific dimensions and poor coverage of psychosocial‐related domains [25]. Alternative instruments, such as the Fatigue Impact Scale (FIS) or the HIV Symptom Index (HSI), have demonstrated greater suitability for this purpose [9]. Specific hypotheses on the role of ART switch in QoL in people with HIV need to be addressed and should align with the choice of a more appropriate QoL instrument [24] in future studies. Nevertheless, the high SF‐36 scores observed in our study confirm the good health status of the virologically suppressed HIV participants included.

Altogether, despite the absence of a control group and limited sample size, these findings underscore the link between treatment satisfaction, treatment durability and consequence maintenance of virologic suppression and highlight the need for patient‐centred care.

## AUTHOR CONTRIBUTIONS

*Conceptualization:* Philippe Mariot. *Methodology:* Bruno Spire; Olivier Robineau; Valérie Pourcher; Philippe Mariot; Jean‐Jacques Parienti; Laurence Slama. *Investigation:* Olivier Robineau; Valérie Pourcher; Paul Loubet; Christia Palacios; Christine Jacomet; Laurence Slama. *Writing—original draft preparation:* Bruno Spire. *Writing—review and editing:* Bruno Spire; Olivier Robineau; Valérie Pourcher; Paul Loubet; Christia Palacios; Christine Jacomet; Philippe Mariot; Haydar Benachir; Jean‐Jacques Parienti; Laurence Slama. *Supervision:* Philippe Mariot.

## CONFLICT OF INTEREST STATEMENT

Haydar Benachir and Philippe Mariot are paid employees of MSD. Olivier Robineau, Valérie Pourcher, Jean‐Jacques Parienti and Laurence Slama have scientific expert contracts with MSD. Paul Loubet has a direct contract with MSD, approved by the competent French authority, to conduct the DoraVIH study.

## Data Availability

The data that support the findings of this study are available from the corresponding author upon reasonable request.
